# Além do Escore de Cálcio para Estratificação de Risco – O Uso do Índice de Carga Inflamatória para Identificar o Doente Vulnerável

**DOI:** 10.36660/abc.20250707

**Published:** 2026-03-13

**Authors:** Diogo Santos-Ferreira

**Affiliations:** 1 Gaia/Espinho Local Health Unit – Cardiology Department Vila Nova de Gaia Porto Portugal Gaia/Espinho Local Health Unit – Cardiology Department, Vila Nova de Gaia, Porto – Portugal; 2 University of Porto Faculty of Medicine Department of Surgery and Physiology Porto Portugal RISE-Health, Department of Surgery and Physiology, Faculty of Medicine, University of Porto, Porto – Portugal

**Keywords:** Aterosclerose, Doença Arterial Coronariana, Inflamação, Fatores de Risco de Doenças Cardíacas

As doenças cardiovasculares (DCV) continuam sendo a principal causa de anos de vida ajustados por incapacidade e de morte em todo o mundo, sendo a doença cardíaca isquêmica a principal responsável.^1^ A identificação oportuna e o controle preciso dos fatores de risco são fundamentais para minimizar o risco cardiovascular, especialmente entre pessoas com maior risco, ou seja, aquelas que já apresentam marcadores de aterosclerose coronária.^2^

A doença coronariana representa uma condição crônica caracterizada por eventos agudos, principalmente decorrentes da ruptura da placa aterosclerótica e do infarto do miocárdio (IM), agravando o prognóstico, especialmente se não houver reperfusão adequada e oportuna. Assim, pacientes com aterosclerose coronariana permanecem em muito alto risco, justificando a adoção de estratégias preventivas intensivas.

Os avanços no diagnóstico permitiram o diagnóstico não invasivo e a quantificação da aterosclerose coronária (utilizando o Escore de Cálcio Coronariano – CAC) e da doença coronária por meio da angiotomografia computadorizada (angioTC). O CAC é um marcador de risco bem estabelecido para IM.^3-5^ No entanto, esse método ainda é imperfeito, pois muitos pacientes com CAC elevado podem nunca sofrer um evento agudo.^6^ No entanto, outros indivíduos com CAC relativamente baixo (ou mesmo inexistente) ainda correm o risco de desestabilização da placa – particularmente de placas de baixa atenuação parcialmente calcificadas ou não calcificadas – e síndrome coronariana aguda, o que justifica ainda mais a busca por novos marcadores de risco cardiovascular, idealmente considerando aspectos que vão além do processo de calcificação.^7^

Nas últimas décadas, tem havido um foco crescente na inflamação como fator de risco para DCV.^8^ Terapias direcionadas à inflamação têm sido estudadas para reduzir o risco de eventos agudos e algumas apresentam resultados promissores.^8^

Ömür et al., em seu manuscrito publicado recentemente,^9^ estudaram a associação de um marcador simples e não invasivo de inflamação, o Índice de Carga Inflamatória (IBI), com a ocorrência de IM em pacientes com doença coronariana não obstrutiva previamente diagnosticada (lesões <50%) por meio de angioTC, e sua interação com o CAC. O IBI é derivado dos níveis de proteína C-reativa (PCR) multiplicados pela razão neutrófilo/linfócito (RNL), proporcionando uma avaliação da inflamação mais abrangente e estável, e vem sendo cada vez mais utilizado como indicador prognóstico em câncer, doenças cerebrovasculares e, mais recentemente, DCV.^10^

Nesta análise retrospectiva de 1.235 pacientes com doença coronariana não obstrutiva, avaliada por angioTC, com um seguimento médio de três anos, 22,9% sofreram um IM, comprovando que se tratava de uma população de muito alto risco, embora o uso de terapia antiplaquetária e estatinas tenha sido relativamente baixo (cerca de 1:2 e 1:4, respectivamente).^9^ Os autores estudaram a associação e a interação entre o IBI e o CAC com o risco de IM durante o acompanhamento. As curvas ROC forneceram os valores de corte ideais para o IBI (128) e o CAC (102), e tanto o IBI elevado quanto o CAC elevado foram associados independentemente ao risco de IM na análise univariada e multivariada (razão de chances para um IBI elevado foi de 2,129 e para um CAC elevado foi de 1,930). Entre os pacientes com um escore de CAC elevado, um IBI elevado foi associado a um risco maior de eventos (37,9% durante o acompanhamento) em comparação com um IBI baixo (34,1% em três anos), abrindo a possibilidade de estudar mais a fundo esse marcador não invasivo como um preditor prognóstico para pacientes com um escore de CAC elevado.^9^ Isso também nos desafia a abordar estes doentes numa visão menos centrada na placa, e a adotar o conceito de paciente vulnerável, em vez de placa vulnerável. Essas descobertas exploratórias de um estudo retrospectivo unicêntrico lançam a primeira pedra para investigar mais a fundo e procurar validação externa do papel do IBI como uma estratificação de risco adicional.^9^

O risco cardiovascular resulta de uma interação complexa de diferentes mecanismos patológicos e abordá-los de forma isolada levará a estratégias preventivas incompletas e insuficientes. A descoberta de novos marcadores de risco, especialmente aqueles resultantes de vias tradicionalmente negligenciadas, como a inflamação, nos ajudará a identificar os pacientes mais vulneráveis e a desenvolver estratégias personalizadas para preveni-los de eventos cardiovasculares.
