# Supplementary material for: Além do Escore de Cálcio para Estratificação de Risco – O Uso do Índice de Carga Inflamatória para Identificar o Doente Vulnerável
Source: Arq Bras Cardiol. 2026 Mar 13;122(11):e20250707. [Article in Portuguese] doi: 10.36660/abc.20250707 (PMC13155787; doi:10.36660/abc.20250707)
Supplement: Translation

# Translation: Improving Risk Stratification Beyond Coronary Calcium – Adding the Inflammatory Burden Index to Find the Vulnerable Patient

**DOI:** 10.36660/abc.20250707i

**Authors:** Diogo Santos-Ferreira

**Affiliations:** 1 Vila Nova de Gaia Porto Portugal Gaia/Espinho Local Health Unit – Cardiology Department, Vila Nova de Gaia, Porto – Portugal; 2 Porto Portugal RISE-Health, Department of Surgery and Physiology, Faculty of Medicine, University of Porto, Porto – Portugal

**Keywords:** Atherosclerosis, Coronary Artery Disease, Inflammation, Heart Disease Risk Factors

Cardiovascular diseases (CVD) remain the leading contributor to disability-adjusted life years and death worldwide, of which ischemic heart disease is the main cause.^1^ Timely identification and incisive management of risk factors is pivotal to minimize cardiovascular risk, particularly among people who are at higher risk, namely those already exhibiting markers of coronary atherosclerosis.^2^

Coronary disease represents a chronic condition characterized by acute events, mainly driven by plaque rupture and myocardial infarction (MI), worsening the prognosis, particularly if a timely and adequate reperfusion is not obtained. Thus, patients with coronary atherosclerosis remain at very high risk, meriting the adoption of intensive preventive strategies.

Advances in diagnostics have further allowed to provide a non-invasive diagnosis and quantification of coronary atherosclerosis (using Coronary Artery Calcium Score – CAC) and coronary disease through Computed Tomography (CT) coronary angiography. CAC is a well-established risk marker for MI.^3-5^ However, it remains imperfect, as many patients with high CAC may never suffer from an acute event,^6^ but others with rather low (or even no) CAC are still at risk of plaque destabilization – particularly from partially calcified or non-calcified low-attenuation plaques - and acute coronary syndrome, further justifying the search of novel markers of cardiovascular risk, ideally looking beyond calcification process.^7^

Over the past decades, there has been an increasing focus on inflammation as a risk factor for CVD,^8^ and therapies targeting inflammation have been studied to reduce the risk of acute events, and some have promising results.^8^

Ömür et al., in their recently published manuscript,^9^ have studied the association of a simple non-invasive marker of inflammation, the Inflammatory Burden Index (IBI), with the occurrence of acute MI in patients with previously diagnosed non-obstructive coronary disease (lesions <50%) using CT angiography, and its interaction with CAC. IBI is derived from the levels of C-reactive protein (CRP) multiplied by neutrophil/lymphocyte ratio (NLR), providing a more comprehensive and stable inflammation assessment, and is being increasingly used as a prognosticator in cancer, cerebrovascular diseases, and, more recently, CVD.^10^

In this retrospective analysis of 1,235 patients with non-obstructive coronary disease through CT angiography, over a mean follow-up of three years, 22.9% of them suffered an acute MI, proving that this was a very-high-risk population, though the use of antiplatelet therapy and statins was rather low (about 1:2 and 1:4, respectively).^9^ The authors have studied the association and interaction of IBI and CAC with the risk of MI over follow-up. ROC curves provided the optimal cut-off values for IBI (128) and CAC (102), and both high IBI and CAC were independently associated with the risk of MI in the univariate and multivariate analysis (odds ratio for a high IBI was 2.129, and for high CAC was 1.930). Among patients with a high CAC score, a high IBI was associated with a higher risk of events (37.9% over the follow-up) versus a low IBI (34.1% over three years), opening the possibility to further study this non-invasive marker as a prognosticator for patients with a high CAC score.^9^ It also defies us to approach these subjects away from a plaque-centered philosophy, and embrace the concept of the vulnerable patient, rather than the vulnerable plaque. These exploratory findings from a retrospective single-center study cast the first stone to further investigate and search for external validation of the role of IBI as an additive risk stratification.^9^

Cardiovascular risk results from a complex interplay of different pathological mechanisms, and approaching them in an isolated fashion will result in incomplete and insufficient preventive strategies. Undercovering novel markers of risk, especially incorporating those resulting from traditionally overlooked pathways, such as inflammation, will further help us find the most vulnerable patients and, hopefully, develop tailored strategies to successfully prevent them from a cardiovascular event.
